# Huachansu injection inhibits metastasis of pancreatic cancer in mice model of human tumor xenograft

**DOI:** 10.1186/1472-6882-14-483

**Published:** 2014-12-13

**Authors:** Jian-Hua Yin, Xiao-Yan Zhu, Wei-Dong Shi, Lu-Ming Liu

**Affiliations:** Department of Oncology, Second TCM Hospital of Guang Dong province, 60 Heng Fu Road, Guang Zhou, Guang Dong province China; Department of Integrative Oncology, Cancer Hospital, Fudan University, 270 Dong An Road, Shanghai, 200032 China

**Keywords:** Huachansu, Pancreatic cancer, Liver metastasis, VEGF, MMPs, CA19-9

## Abstract

**Background:**

Huachansu injection (HCS) is a water-soluble preparation made from Bufo gargarizans’s skin, which has been widely used in clinics for tumor therapy in China. Though the anti-cancer activity of HCS has been verified through studies in vitro and in vivo, there is little research about its potential anti-metastasis effect. The primary objective of this study was to assess the effects of HCS on both the invasion of pancreatic cancer cells in vitro and on the progression of liver metastasis in vivo in this study.

**Methods:**

HCS anti-metastasis potential was accessed using both assay of Cell viability and invasion in vitro, and then further Establishing xenograft model in nude mice. In the cell-based assay, mRNA and protein expression of MMP-2, MMP-9 and VEGF was detected by semi-quantitative RT-PCR and western blotting. In animal experiment, liver metastasis nodules and change of liver-body ratio was observed. Meanwhile, correlation of the CA19-9 and CEA content in serum with the progression of liver metastasis was analyzed.

**Result:**

We observed that HCS prevented the invasion of cancer cells, with inhibiting the expressions of MMP-2 and MMP-9, and reduced not only the number of metastasis nodules but the ratio of liver-body weight as well. Furthermore, HCS decreased the expression of MMP-2, MMP-9 and VEGF in liver metastasis, while also reducing CA19-9 contents in serum. In addition, correlation analysis indicated that the level of CA19-9 in serum was closely related to the number of liver metastasis nodules.

**Conclusion:**

Our experimental results suggest that HCS has some anti-metastasis potential to suppress the growth of liver metastasis by decreasing the expression of MMP-2 and MMP-9 as well as VEGF.

**Electronic supplementary material:**

The online version of this article (doi:10.1186/1472-6882-14-483) contains supplementary material, which is available to authorized users.

## Background

Pancreatic cancer has been considered an aggressive and depressive malignancy with dismal survival rate [1] because of early metastasis and delayed diagnosis, even after optimal conventional therapy such as curative resection of the primary tumor, radiation and chemo-therapy [2, 3]. It is likely to metastasize early for this disease even when small, except the cancer cells get generally its resistance to chemotherapy and/or radiotherapy [4–6]. It was reported that 80% of these patients had been already in local infiltration and distant metastasis when diagnosed, among which the incidence of liver metastasis was more than 50% [7, 8]. In fact, Metastasis is considered as the primary cause of death in patients with pancreatic carcinoma in recent year [9]. For all these reasons, there has been calling for novel agents and therapeutic strategies for this disease.

The detailed mechanisms of how pancreatic cancer cells spread are poorly understood. However, behavior of malignant cells is closely linked to changes in their microenvironment [10], in which stroma is an important component .In recent years, Importance of tumor stroma in facilitating tumor cell growth and invasion has been more clearly defined [11]. Vascular endothelial growth factor (VEGF), Matrix metalloproteinases (MMPs) including MMP-2 and MMP-9 are abundant in the stroma around malignant pancreatic tumor [12, 13]. MMPs are believed to be important facilitators of cancer cell invasion in past studies by resolving basement membrane proteins, Because of rich MMP-2 production found in the stromal cells of pancreatic cancer [14] and the expression of MMP-9 increasing in adjacent pancreatic cancer cells [15]. VEGF is strongly induced by hypoxia forming within the tumor microenvironment and VEGF keeps not only its role as the most critical driver of vascular formation but also stimulates the invasion and metastasis of tumor cells [16, 17].

HCS is a water-soluble preparation made from Bufo gargarizans’s skin, which has been widely used in China and other Asian countries in clinics for malignant tumors [18, 19]. So far, it has been verified that HCS has significant anti-cancer effects in some gastrointestinal tumors such as hepatic, gastric, pancreatic and esophageal carcinoma [20–23]. Experiment showed that anti-tumor effects of HCS has been observed in Human Pancreatic Cancer-cell mice, with the increase in concentration of HCS, cell cycle was maintained at the S-phase, with pRb protein expression upregulation [24, 25]. Recently, studies in clinic have suggested that HCS alone or combined with other chemotherapeutic agents, effectively improves the survival rate and the quality of life for patients with gastrointestinal tumors such as gallbladder, liver and pancreatic cancer [26–28]. In addition, in a phase trials sponsored by our department and M.D. Anderson Cancer Center, no dose-limiting toxicity (DLT) was observed with the use of HCS at doses up to 8 times higher than typically used in China and six patients had stable disease prolonged or minor tumor shrinkage [29].

However, it has not been clarified whether HCS has anti-metastasis effects on advanced pancreatic cancer. The purpose of this study is to evaluate the anti-metastasis activity of HCS and further to explore its underlying anti-metastasis mechanisms.

## Methods

### Cell lines and mice

Human pancreatic cancer cell lines SW1990, BxPC3 and CF-PAC1 were obtained from the American Type Culture Collection and grown in complete growth medium as recommended by the manufacturer. The cultured cells were maintained in a humidified 5% CO2 atmosphere at 37. Female BALB/c-nu/nu nude mice (18-22 g) were obtained from Shanghai Laboratory Animal Center Chinese Academy of Sciences (Shanghai, China) and housed in laminar flow cabinets under specific pathogen-free conditions with food and water ad libitum. The study protocol was approved by the Shanghai Medical Experimental Animal Care Committee.

### Drugs and reagents

HCS, prepared by concentrating the extraction of toad skin 10 g to 1 ml, was obtained from Anhui Jinchan Biochemical Co., Ltd. (Anhui, China). RPMI-1640 and fetal bovine serum were purchased from Gibco (U.S.A). VEGF monoclonal antibody was from BD Pharmingen (U.S.A); MMP-9 and MMP-2 monoclonal antibody were from Santa Cruz biotechnology, inc.(U.S.A). Cell counting kit-8(CCK-8) was purchased from Dojindo, Molecular Technologies, inc. (Gaithersburg, MD, U.S.A).

### Cell viability assay

Briefly, cells were suspended at a final concentration of 5 × 10^3^ cells/well and cultured in triplicate in a 96-well microplate. After exposure to HCS, the viability of cells was measured using the CCK-8 assay at 36 h, 48 h and 72 h. Then, CCK-8(10 μl) was added to each well containing 100 μl mixture of culture medium and HCS (0, 0.156, 0.312, 0.625, 1.25, 2.50, 5.0 mg/ml, respectively). The plate was incubated for 1 h at 37°C. Viable cells were counted by absorbance measurements at 450 nm using auto microplate reader (Tecan Sunrise, Austria). The OD450 value was proportional to the viability of cell. All experiments were performed in triplicate on three separate occasions.

### Cell invasion assay

Transwell chamber invasion assay was used to test the effect of HCS on in vitro invasion of SW1990 cells according to the manufacturer’s advice (BD Biosciences). In brief, 500 μl of serum free media containing 1.0 × 10^5^ SW1990 cells treated with or without HCS (10, 20 and 50ug/ml) were added into the upper chamber in duplicate and allowed to invade toward the lower chamber with 10% fetal bovine serum (FBS). After incubation for 24 hrs, the cells on the upper side of the chambers were removed with a cotton swab and the cells on the lower surface were fixed in 95% ethanol and stained with 0.1% crystal violet. Then, the transmigrated cells were counted. For each experiment, 10 random high power fields were chosen as described previously [30].

### Reverse transcription polymerase chain reaction analysis

Total RNA was extracted by TRIzol reagent (Invitrogen, San Diego, CA, USA) from SW1990 cells treated for 24 hr with HCS of 50ug/ml. 2 μg of purified total RNA was used for reverse transcription PCR (RT-PCR) according to the manufacturer’s instructions (MBI Fermentas, Vilnius, Lithuania). Sequences of the PCR primers were: VEGF forward 5′-CTACCTCCACCATGCCAAGT-3′, reverse 5′-TCTCTCCTATG TGCTGGCCT-3′; MMP-2 forward 5′-CAGGCTCTTCTCCTTTCACAAC-3′, reverse 5′-AAGCCACG GCTTGGTTTTCCTC-3′; MMP-9 forward 5′-GGCGCTCATGTACCC TATGT-3′, reverse 5′-CTCAAA GACCGAGTCCAGCT-3′; GAPDH forward 5′-GGGAGCCAAAAGGGT CATCATCTC-3′, reverse 5′-CCATGCCAGTGAGCTTCCCGTTC-3′ chosen as an internal control. We used Quantity-One Gel Imaging software (BIO-RAD) to perform quantitative RT-PCR analyses of mRNA levels, relative to GAPDH levels. All primers were synthesized at Shanghai Sangon Biological Engineering & Technology Services Co (Shanghai, China).

### Western blot analysis

After treatment with 50ug/ml HCS for 24 h, cells were harvested and lysed in ice-cold NP40 lysis buffer with protease inhibitor (0.5 mM phenylmethylsulfonyl fluoride, PMSF) for 30 minutes on ice. Then the lysates were centrifuged at 12,000 rpm for 15 minutes and the supernatant was collected. Equal amounts of protein (30 μg) were separated by 10% SDS-PAGE and then incubated with anti-human monoclonal antibodies. Protein bands were visualized by the enhanced chemiluminescence (ECL) kit (Amersham Pharmacia Biotech, Uppsala, Sweden). Images were analyzed with ImageJ software supported by National Institute of Health (NIH).

### Establishment of xenograft model in nude mice

6 × 10^6^ in 0.2 mL of SW1990 cells in logarithmic phase were inoculated into mice right armpit. Mice were sacrificed and then the subcutaneous tumor was removed when the tumor grew approximately 1000 mm3 in volume. The tumor tissue was sheared and then ground into cell suspensions of 3 × 10^7^/mL at final concentration in serum-free PBS (Phosphate Buffered Saline). Cell viability greater than 95% was determined by trypan blue exclusion. The mice were anesthetized with 10% chloral hydrate and the spleen was exteriorized through a left flank incision. 3 × 10^6^ cells in 100 μL of suspensions were slowly injected into the splenic pulp using an inoculating needle. The spleen was relocated into the abdominal cavity and then the peritoneum and skin were closed. All animals tolerated the procedure well.

### Animal administration and assessment of liver metastasis in vivo

A total of 27 mice with intrasplenic inoculation were randomly divided into three groups a week later: Control group administered with normal saline, HCS-L group and HCS-H group administered with HCS at a dose of 1500 mg/Kg/day and 3000 mg/Kg/day separately. Each group consisted of 9 mice. The daily experimental dosage was calculated according to the following human–mouse transfer formula: Db = Da × Rab, where Da, Db and Rab represent human dosage (g/kg), mouse dosage (g/kg), and conversion coefficient respectively. The typical dose of HCS used for patients in China is 10-15 g daily (approximately 7.5 g/m2). All mice received intraperitoneal injection of 0.4 ml normal saline or HCS each day after randomized division. Mice were weighed each week. All experiments on mice were conducted in accordance with the guidelines of the NIH for the care and use of laboratory animals. Mice were sacrificed six weeks after inoculation and then blood sampling was collected through mouse eye’s blood vessel. The blood samples were centrifuged and the serums were collected for ELISA assay. The livers were excised and weighed for assessing the ratio of liver to body weight (g/100 g), and the metastases on the surface of liver were enumerated by a dissecting microscope. Liver metastases were placed in 10% neutral buffered formalin for immunohistochemistry.

### Immunohistochemical analysis

Liver metastases in 10% buffered formalin were dehydrated and embedded in paraffin. Dewaxed paraffin-embedded sections measured 4 μm were immunostained according to the protocol of streptavidin-peroxidase (SP) kit (ZYMED, U.S.A). Immunoreactions were visualized with HRP conjugated IgG (goat anti-rabbit/mouse antibody) and developed in diaminobenzidine. The sections were counterstained with hematoxylin and permanently mounted. The slides in 10 randomly selected high power fields (HPF, 400×) were examined in a blinded fashion with light microscopy by two independent researchers [31]. ImageJ software was used to analyze the optical density (OD) of positively-stained cells on the slides.

### Enzyme-linked immunosorbent assay (ELISA) of serum samples

Of the corresponding liver metastasis samples, carbohydrate antigen CA19-9 and carcinoembryonic antigen (CEA) levels were measured using Human Immunoassay kits (Calbiotech, CA, USA) in 19/27 serum samples available from the mice with liver metastasis. Serum samples were assayed in duplicate on two separate occasions with associated standards as controls in accordance with manufacturer’s recommendations.

### Statistical analysis

Group differences were statistically analyzed using Student’s *t*-test or One-way analysis of variance for unpaired values, Mann-Whitney’s *U* test was used to analyze the metastases in liver, Kruskal-Wallis test for the ratio of liver to body weight, and Correlation of the CA19-9 and CEA content in serum with the liver metastasis was evaluated by Linear Regression test. The level of significance was set at p < 0.05, and data are expressed as mean ± standard deviation (SD). The statistical analysis was performed using GraphPad prism version 5.01 software (SanDiego, CA, USA).

## Results

### HCS inhibited pancreatic cells viability and the invasion of SW1990 cells

**Figure 1:**
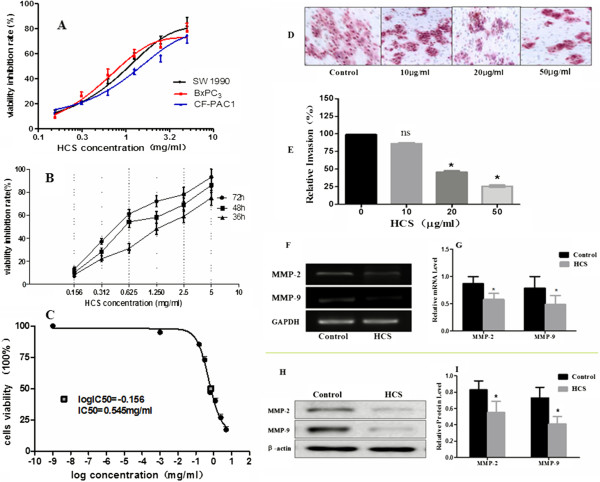
**Inhibitory effects of HCS on the viability and invasive behavior of pancreatic cancer cells. (A)** Effects of HCS on proliferation of pancreatic cancer cell lines (SW1990, BxPC_3_ and CF-PAC1) after 48 hours. **(B)** Effects of HCS on proliferation of SW1990 cells after 36, 48 and 72 hours. **(C)** IC50=0.545mg/ml was calculated in the time-dose-curve at 48 hour for SW1990. **(D)** After 24 hours of incubation, cells that migrated to the lower side of the chamber were stained with 1% crystal violet and counted under a microscope. **(E)** The invasive capability of SW1990 cells was reduced in a dose-dependent manner. **(F-I)** 24 hours after HCS (50μg/ml) treatment, the mRNA expression of MMP-2 and MMP-9 was reduced by 32.8±2.6% and 37.4±2.8% respectively **(F,G)**; the protein expression of decreased by 33.7±2.5% and 43.8±3.7% respectively **(H,I)**, as compared to the Control (P<0.05). Data are from three independent experiments. In the bar graph, *: P<0.05 indicates the difference significant statistically, compared to controls; “ns” presents no significant difference.

To examine whether treatment with HCS influences proliferation of tumor cells, we employed CCK-8 assays. The cells of three cell lines (SW1990, BxPC3 and CF-PAC1) were treated with HCS of different concentrations (0.156, 0.312, 0.625, 1.25, 2.5 and 5.0 mg/ml). After 48 hours, cell growth was inhibited in all cell lines (Figure 1A). Since there was no standard concentration for the use of HCS in cell experiments, we used several different concentrations mentioned above to test the effect of HCS on the growth of SW1990 cells at various time points (36, 48 and 72 h). The inhibition rate was 8%-75% at 36 h, 10%-86% at 48 h and 13%-96% at 72 h respectively; The higher the concentration of HCS, the better the effect of its inhibition (Figure 1B). The inhibition effect of 0.156 mg/ml was not different among at 36 h, 48 h and 72 h (P > 0.05), but in other concentrations we found the inhibition effect at 48 h was higher than at 36 h (P < 0.05), 72 h higher than 48 h (P < 0.05), Differences between two groups were evaluated by *t*-test analysis. P < 0.05 was considered statistically significant. We chose the IC50 value (50% inhibitory concentration) at 48 h as the reference indicators in this experiment. According to CCK-8 introduction, IC50 value (50% inhibitory concentration) was calculated to be about 0.545 mg/ml at 48 h (Figure 1C).In order to further determine the effect of HCS on the invasive behavior of tumor cells, we adopted transwell test. SW1990 cells with highly-metastatic potential were treated with HCS of 0.01, 0.02 and 0.05 mg/ml respectively. After 24 hours, HCS significantly inhibited the invasion of SW1990 cells at the concentration of 0.02 mg/ml and 0.05 mg/ml (P < 0.05), as compared with the control untreated (Figure 1D,E).

### HCS reduced the expressions of MMP-2 and MMP-9 in SW1990 cells in vitro

As is well known, MMP-2 and MMP-9 have been implicated in metastasis and invasion of malignant tumor cells [32]. Here, semi-quantitative RT-PCR and western blotting were performed for MMP-2 and MMP-9 in SW1990 cells. The bands of MMP-2 and MMP-9 at mRNA and protein levels indicated significant difference between the Control and HCS groups (Figure 1F,G,H,I). The protein expression of MMP-2 and MMP-9 decreased by 33.7 ± 2.5% and 43.8 ± 3.7% respectively; the mRNA expression of them reduced by 32.8 ± 2.6% and 37.4 ± 2.8% respectively in HCS-L/H groups, compared to the Control (P < 0.05).

### HCS inhibited the progression of liver metastasis in vivo

**Figure 2:**
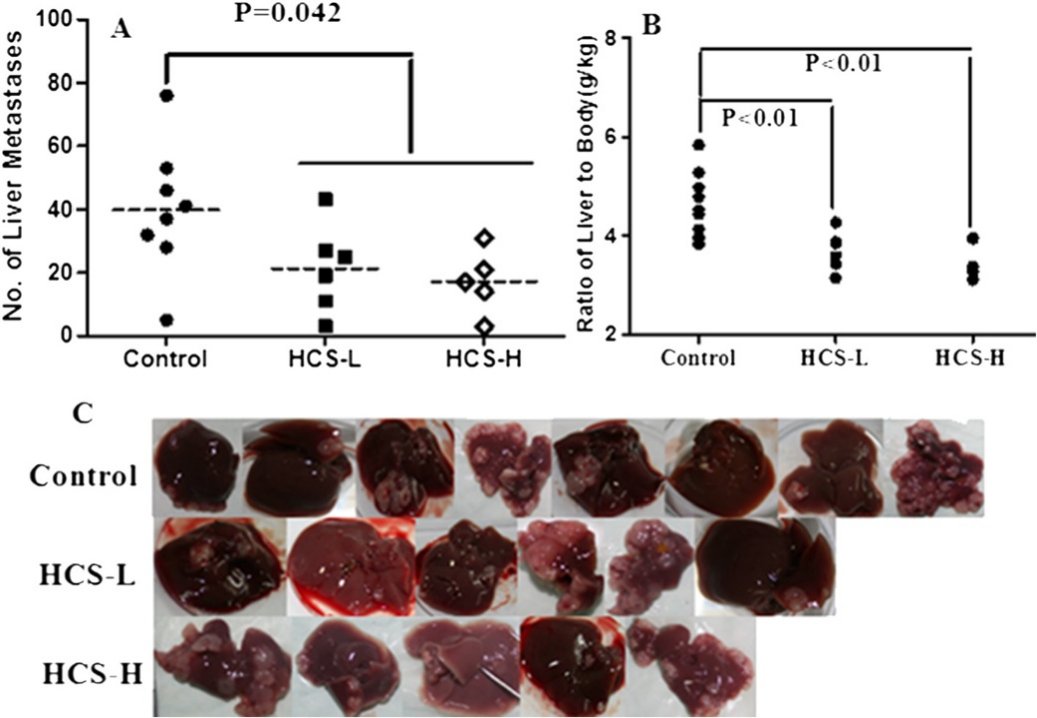
**Effect of HCS on the liver metastases from human pancreatic cancer by SW1990 cells. (A)** The number of liver metastases in HCS-treated groups was less than the Control (P = 0.042). **(B)** The ration of liver-body weight was decreased in HCS-treated groups, compared to the Control (P<0.01). **(C)** Livers with metastatic nodules in Control (8/9), HCS-L (6/9) and HCS-H (5/9) groups.

To further observe the effects of HCS on tumor metastasis in vivo, we established an animal model with liver metastasis from human pancreatic cancer by SW1990 cells. After HCS treatment described above, experimental animals were weighed and then sacrificed. At the same time, livers were excised and then weighed. We identified liver metastases in 8 of 9 mice in Control-group, 6 of 9 in HCS-L group and 5 of 9 in HCS-H group respectively (Figure 2C). It was observed that there was a dramatic reduction in the number of liver metastases as well as in the ratio of liver to body weight in the HCS-treated animals (Figure 2A,B). The average number of metastatic tumors per liver was 35.7 ± 4.2, 19.4 ± 2.8 and 14.5 ± 2.3and the ratio of liver to body weight was 4.64 ± 0.28 g/100 g, 3.72 ± 0.16 g/100 g and 3.53 ± 0.17 g/100 g (P < 0.05, seen in Additional file 1: Table S1), corresponding to the Control, HCS-L and HCS-H treated group respectively. All these evidences indicated that HCS could inhibit the progression of liver metastasis from pancreatic cancer.

### HCS reduced the expressions of VEGF, MMP-2 and MMP-9 in metastasis in vivo

**Figure 3:**
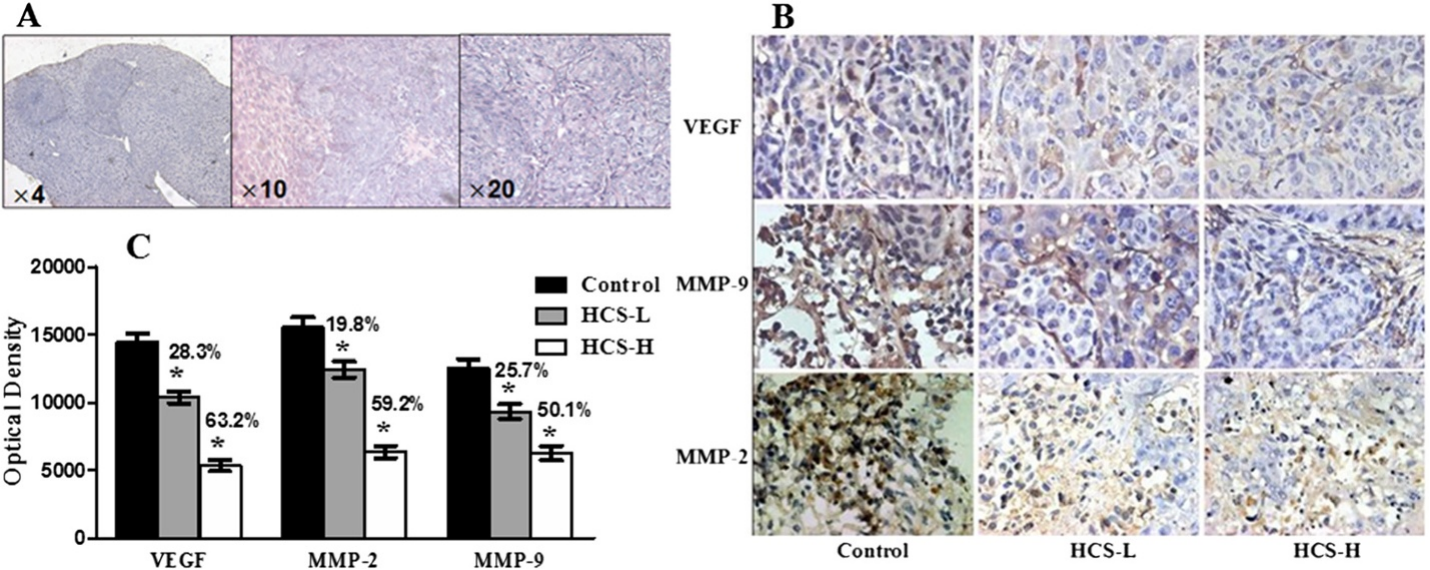
**Immunohistochemical analysis of VEGF, MMP-2 and MMP-9 expression in liver metastases. (A)** H-E staining of liver metastasis ×4, 10, 20 magnification; **(B)** Detection for the expression of VEGF, MMP-2 and MMP-9 by streptavidin-peroxidase (SP) conjugated method, ×40 magnification; **(C)** With optical density analysis of liver tumor sections by ImageJ software, the expression of VEGF was reduced by 28.3±2.2% and 63.2±5.7%, MMP-2 by 19.8±1.7% and 59.2±6.3% as well as MMP-9 by 25.7±2.4% and 50.1±5.2% in HCS-L and HCS-H groups respectively, compared to the Control (P<0.05), by Mann–Whitney test. Data is mean±SD.

To determine the possible suppression pathway of HCS to metastatic mechanism, we detected the expression of VEGF, MMP-2 and MMP-9 in liver metastases using immunohistochemistry analysis. All immunostained tumor sections were examined by bright-field microscopy and photographed with a Nikon ECLIPSE E600 microscope (Nikon, Japan) camera using 4×,10×, 20× and 40× objective (Figure 3A,B). Images were loaded into the ImageJ software for the optical density (OD) analysis as described previously [33]. Densitometric analysis exhibited that the expression of VEGF, MMP-2 and MMP-9 in liver metastases was reduced by 28.3%, 19.8% and 25.7% respectively in the HCS-L group, and decreased by 63.2%, 59.2% and 50.1% separately in the HCS-H group, as compared with the Control (Figure 3C).

### HCS impacts on the concentration of CEA and CA19-9 in serum

**Figure 4:**
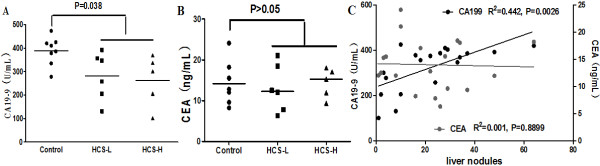
**ELISA analysis for the concentration of CEA and CA19-9 in serum after HCS treatment. (A)** The CA19-9 concentrations in serum in HCS-treated groups differed significantly from that of the Control (P = 0.038); **(B)** The CEA concentrations were not different statistically among the Control and HCS groups (P>0.05), Data analyzed by Kruskal-Wallis test. **(C)** The linear regression analysis indicated that after HCS treatment, CA19-9 in serum decreased with the number of liver metastases reduced (R^2^ = 0.442, P = 0.0026), but which was independent of CEA in serum (R^2^ = 0.001, P = 0.8899).

As is well known, CA19-9 is a very important prognostic factor in pancreatic cancer. In this experiment, we detected the concentration of CEA and CA19-9 in serum by ELISA assay in 19 blood samples. The CA19-9 concentration in HCS-treated groups (mean was 281.5U/ml and 262.8U/ml in HCS-L and HCS-H groups respectively) differed significantly from that of the Control (388.1U/ml) (Figure 4A; P = 0.038). Meanwhile, there was not any dramatical difference in the contents of serum CEA among three experimental groups, though the CEA concentration was decreased in HCS groups (Figure 4B; P > 0.05).

### Correlation of the CA19-9 and CEA content in serum with the progression of liver metastasis

Subsequently, we evaluated the relation between the content of CA19-9, CEA in serum and the number of liver metastatic nodules so as to observe the HCS influence on progression of advanced pancreatic cancer. Linear regression analysis indicated that CA19-9 content in serum was related positively with the number of liver nodules (R2 = 0.442, P = 0.0026), which was yet not linked obviously with CEA content in serum (R2 = 0.001, P = 0.8899) (Figure 4C).

## Discussion

Pancreatic cancer remains an unfortunate disease with a 5-year survival rate below 6%, a worst survival in all cancers [34] for its early remote dissemination. This reflects the inability of current systemic therapy to prevent or treat pancreatic cancer metastases and underscores the need for novel therapy.

The research of HCS has been going on in America, China and some other Asian countries such as Japan and Korea. Over recent years, HCS is gaining ever-increasing attention due to its various biological activities displayed in therapy of many diseases [35, 36]. In this study, we tried to examine whether HCS had some anti-metastasis effects on pancreatic cancer. In vitro HCS evidently inhibited the invasion of SW1990 cells as well as the cell viability of three cell lines (SW1990, BxPC3 and CF-PAC1), and in vivo the number of liver metastases was decreased, accompanied with the ratio of liver-body weight improved by HCS in the nude mice model. It means that HCS has the potential of anti-metastasis effect on advanced pancreatic cancer.

Studies of matrix metalloproteases (MMPs) in invasive cancers have indicated that MMPs play an important role in the development of tumor metastases, among which MMP-2 and MMP-9 are particularly associated with distant dissemination of malignant tumors [37]. Moreover, some researches were reported that high expression of MMP-2 and MMP-9 was found in pancreatic cancer [38, 39]. Our findings showed that the expressions of MMP-2 and MMP-9 at the levels of mRNA and protein were significantly down-regulated by HCS treatment, indicating that HCS inhibited the growth of liver metastasis possibly through decreasing the expression of MMP-2 and MMP-9.

Angiogenesis depends on numerous activating factors that were demonstrated to be over-expressed in pancreatic cancer, while it has been verified that VEGF is a key inducer of angiogenesis. Over recent years, VEGF has been targeted in clinic for treatment of some cancers such as rectal cancer, lung cancer and liver cancer [40–42]. In this experiment, HCS abated the expression of VEGF in liver metastasis, which implied that maybe there was some relationship between VEGF and MMPs in the context of this experiment. This result was also consistent with recent studies that MMPs enhanced tumor angiogenesis by triggering the angiogenic switch [43].

As a prognostic marker, the serum CA19-9 has replaced CEA because it is more specific and sensitive for pancreatic cancer [1, 44]. In this experiment, the CA19-9 content in serum declined obviously from 388.1U/ml in the Control to 262.8-281.5U/ml in HCS-treated animals (data not shown); however, the concentration of CEA in serum changed little among the Control and HCS groups. Incidentally, there was no dramatic difference in terms of CA19-9 between the low and high doses of HCS, corresponding to the results above that the number of liver metastases showed no difference between HCS groups. Then, linear regression analysis indicated that the change of CA19-9 was closely related with the number of liver metastasis, which confirmed that CA19-9 in serum reflected the progression of advanced pancreatic cancer with liver metastasis as a prognostic maker.

## Conclusion

In this study, based on the present findings, we inferred that HCS could inhibit the progression of advanced pancreatic cancer with liver metastasis through down-regulating the expression of VEGF, MMP-2 and MMP-9. Nevertheless, this experiment only throws some light on the mechanism of HCS inhibitory effects on metastasis and progression of advanced pancreatic cancer, and so more researches should be pushed on in further studies.

## Electronic supplementary material

Additional file 1: Table S1: Effect of HCS treatment on the progression of liver metastasis from advanced pancreatic cancer. (DOC 25 KB)

## References

[1] Jemal A, Siegel R, Ward E, Hao Y, Xu J, Murray T, Thun MJ (2008). Cancer statistics, 2008. CA Cancer J Clin.

[2] Lundin J, Roberts PJ, Kuusela P, Haglund C (1994). The prognostic value of preoperative serum levels of CA 19-9 and CEA in patients with pancreatic cancer. Br J Cancer.

[3] Breslin TM, Hess KR, Harbison DB, Jean ME, Cleary KR, Dackiw AP, Wolff RA, Abbruzzese JL, Janjan NA, Crane CH, Vauthey JN, Lee JE, Pisters PW, Evans DB (2001). Neoadjuvant chemoradiotherapy for adenocarcinoma of the pancreas: treatment variables and survival duration. Ann Surg Oncol.

[4] DiMagno EP, Reber HA, Tempero MA (1999). AGA technical review on the epidemiology, diagnosis, and treatment of pancreatic ductal adenocarcinoma, American Gastroenterological Association. Gastroenterology.

[5] Sener SF, Fremgen A, Menck HR, Winchester DP (1999). Pancreatic cancer: a report of treatment and survival trends for 100,313 patients diagnosed from 1985-1995, using the National Cancer Database. J Am Coll Surg.

[6] Kornmann M, Beger HG, Link KH (2003). Chemosensitivity testing and test-directed chemotherapy in human pancreatic cancer. Recent Results Cancer Res.

[7] Fortner JG (1984). Regional pancreatectomy for cancer of the pancreas, ampulla, and other related sites. Tumor staging and results. Ann Surg.

[8] Smeenk HG, Incrocci L, Kazemier G, van Dekken H, Tran KT, Jeekel J, van Eijck CH (2005). Adjuvant 5-FU-based chemoradiotherapy for patients undergoing R-1/R-2 resections for pancreatic cancer. Dig Surg.

[9] Stupack DG, Cheresh DA (2002). ECM remodeling regulates angiogenesis: endothelial integrins look for new ligands. Sci STKE.

[10] Bissell MJ, Radisky D (2001). Putting tumours in context. Nat Rev Cancer.

[11] Bhowmick NA, Moses HL (2005). Tumor-stroma interactions. Curr Opin Genet Dev.

[12] Muerkoster S, Wegehenkel K, Arlt A, Witt M, Sipos B, Kruse ML, Sebens T, Kloppel G, Kalthoff H, Folsch UR, Schafer H (2004). Tumor stroma interactions induce chemoresistance in pancreatic ductal carcinoma cells involving increased secretion and paracrine effects of nitric oxide and interleukin-1beta. Cancer Res.

[13] Teraoka H, Sawada T, Nishihara T, Yashiro M, Ohira M, Ishikawa T, Nishino H, Hirakawa K (2001). Enhanced VEGF production and decreased immunogenicity induced by TGF-beta 1 promote liver metastasis of pancreatic cancer. Br J Cancer.

[14] Maatta M, Soini Y, Liakka A, Autio-Harmainen H (2000). Differential expression of matrix metalloproteinase (MMP)-2, MMP-9, and membrane type 1-MMP in hepatocellular and pancreatic adenocarcinoma: implications for tumor progression and clinical prognosis. Clin Cancer Res.

[15] Qian X, Rothman VL, Nicosia RF, Tuszynski GP (2001). Expression of thrombospondin-1 in human pancreatic adenocarcinomas: role in matrix metalloproteinase-9 production. Pathol Oncol Res.

[16] Grzeszkiewicz TM, Lindner V, Chen N, Lam SC, Lau LF (2002). The angiogenic factor cysteine-rich 61 (CYR61, CCN1) supports vascular smooth muscle cell adhesion and stimulates chemotaxis through integrin alpha (6) beta (1) and cell surface heparan sulfate proteoglycans. Endocrinology.

[17] Babic AM, Kireeva ML, Kolesnikova TV, Lau LF (1998). CYR61, a product of a growth factor-inducible immediate early gene, promotes angiogenesis and tumor growth. Proc Natl Acad Sci U S A.

[18] Chen KK, Kovarikova A (1967). Pharmacology and toxicology of toad venom. J Pharm Sci.

[19] Zhang J, Sun Y, Liu JH, Yu BY, Xu Q (2007). Microbial transformation of three bufadienolides by Nocardia sp. and some insight for the cytotoxic structure-activity relationship (SAR). Bioorg Med Chem Lett.

[20] Gan T, Wu Z, Tian L, Wang Y (2010). Chinese herbal medicines for induction of remission in advanced or late gastric cancer. Cochrane Database Syst Rev.

[21] Chen Z, Zhai XF, Su YH, Wan XY, Li J, Xie JM, Gao B (2003). Clinical observation of cinobufacini injection used to treat moderate and advanced primary liver cancer. Zhong Xi Yi Jie He Xue Bao.

[22] Wang J, Jin Y, Xu Z, Zheng Z, Wan S (2009). Involvement of caspase-3 activity and survivin downregulation in cinobufocini-induced apoptosis in A 549 cells. Exp Biol Med (Maywood).

[23] Qi F, Li A, Inagaki Y, Xu H, Wang D, Cui X, Zhang L, Kokudo N, Du G, Tang W (2012). Induction of apoptosis by cinobufacini preparation through mitochondria- and Fas-mediated caspase-dependent pathways in human hepatocellular carcinoma cells. Food Chem Toxicol.

[24] Xiao-yan Z, Zhi-qiang M, Zhen C, Jun-hua L, Ye-hua S, Wun A, Peng W, Jian-qin H, Lu-ming L (2009). Anti-tumor Effects ofD ifferentFractions from C inobutacini in human pancreatic cancer-bearing SW1990 mice. SHJTCM.

[25] Xiao-yan Z, Lu-ming L, Zhen C, Jun-hua L, Li-tao X, Zhi-qiang M (2013). Effects of “Huachansu Injecion” combined with Gemcitabine on PANC-1 cell proliferation and cell cycle. SHJTCM.

[26] Qin TJ, Zhao XH, Yun J, Zhang LX, Ruan ZP, Pan BR (2008). Efficacy and safety of gemcitabine-oxaliplatin combined with huachansu in patients with advanced gallbladder carcinoma. World J Gastroenterol.

[27] Zuo X, Cui Y (2003). Clinical research progress on the antitumor effects of cinobufacini. China Clin Oncol.

[28] Hai-tao D, Yong-he HE (2007). Therapeutic effects of the combination of cinobufacini injection and chemotherapy in big artery for 130 patients with middle and late stages of pancrea cancer. China J New Drugs.

[29] Meng Z, Yang P, Shen Y, Bei W, Zhang Y, Ge Y, Newman RA, Cohen L, Liu L, Thornton B, Chang DZ, Liao Z, Kurzrock R (2009). Pilot study of huachansu in patients with hepatocellular carcinoma, nonsmall-cell lung cancer, or pancreatic cancer. Cancer.

[30] Subramanian G, Schwarz RE, Higgins L, McEnroe G, Chakravarty S, Dugar S, Reiss M (2004). Targeting endogenous transforming growth factor beta receptor signaling in SMAD4-deficient human pancreatic carcinoma cells inhibits their invasive phenotype1. Cancer Res.

[31] Juuti A, Lundin J, Nordling S, Louhimo J, Haglund C (2006). Epithelial MMP-2 expression correlates with worse prognosis in pancreatic cancer. Oncology.

[32] Nelson AR, Fingleton B, Rothenberg ML, Matrisian LM (2000). Matrix metalloproteinases: biologic activity and clinical implications. J Clin Oncol.

[33] Ruifrok AC, Johnston DA (2001). Quantification of histochemical staining by color deconvolution. Anal Quant Cytol Histol.

[34] Jemal A, Siegel R, Xu J, Ward E (2010). Cancer statistics, 2010. CA Cancer J Clin.

[35] Hong Z, Chan K, Yeung HW (1992). Simultaneous determination of bufadienolides in the traditional Chinese medicine preparation, liu-shen-wan, by liquid chromatography. J Pharm Pharmacol.

[36] Bick RJ, Poindexter BJ, Sweney RR, Dasgupta A (2002). Effects of Chan Su, a traditional Chinese medicine, on the calcium transients of isolated cardiomyocytes: cardiotoxicity due to more than Na, K-ATPase blocking. Life Sci.

[37] Kleiner DE, Stetler-Stevenson WG (1999). Matrix metalloproteinases and metastasis. Cancer Chemother Pharmacol.

[38] Gress TM, Muller-Pillasch F, Lerch MM, Friess H, Buchler M, Adler G (1995). Expression and in-situ localization of genes coding for extracellular matrix proteins and extracellular matrix degrading proteases in pancreatic cancer. Int J Cancer.

[39] Nagakawa Y, Aoki T, Kasuya K, Tsuchida A, Koyanagi Y (2002). Histologic features of venous invasion, expression of vascular endothelial growth factor and matrix metalloproteinase-2 and matrix metalloproteinase-9, and the relation with liver metastasis in pancreatic cancer. Pancreas.

[40] Willett CG, Duda DG, di Tomaso E, Boucher Y, Ancukiewicz M, Sahani DV, Lahdenranta J, Chung DC, Fischman AJ, Lauwers GY, Shellito P, Czito BG, Wong TZ, Paulson E, Poleski M, Vujaskovic Z, Bentley R, Chen HX, Clark JW, Jain RK (2009). Efficacy, safety, and biomarkers of neoadjuvant bevacizumab, radiation therapy, and fluorouracil in rectal cancer: a multidisciplinary phase II study. J Clin Oncol.

[41] Sandler A, Gray R, Perry MC, Brahmer J, Schiller JH, Dowlati A, Lilenbaum R, Johnson DH (2006). Paclitaxel-carboplatin alone or with bevacizumab for non-small-cell lung cancer. N Engl J Med.

[42] Salmon JS, Lockhart AC, Berlin J (2005). Anti-angiogenic treatment of gastrointestinal malignancies. Cancer Invest.

[43] Bergers G, Brekken R, McMahon G, Vu TH, Itoh T, Tamaki K, Tanzawa K, Thorpe P, Itohara S, Werb Z, Hanahan D (2000). Matrix metalloproteinase-9 triggers the angiogenic switch during carcinogenesis. Nat Cell Biol.

[44] Pleskow DK, Berger HJ, Gyves J, Allen E, McLean A, Podolsky DK (1989). Evaluation of a serologic marker, CA19-9, in the diagnosis of pancreatic cancer. Ann Intern Med.

[CR45] The pre-publication history for this paper can be accessed here:http://www.biomedcentral.com/1472-6882/14/483/prepub

