# Supplementary material for: Huachansu injection inhibits metastasis of pancreatic cancer in mice model of human tumor xenograft
Source: BMC Complement Altern Med. 2014 Dec 13;14:483. doi: 10.1186/1472-6882-14-483 (PMC4320457; doi:10.1186/1472-6882-14-483)
Supplement: Supplementary file 1 — Additional file 1: Table S1: Effect of HCS treatment on the progression of liver metastasis from advanced pancreatic cancer. (DOC 25 KB) [file 12906_2013_2083_MOESM1_ESM.doc]

Table Effect of HCS treatment on the progression of liver metastasis from advanced pancreatic cancer

Group No. of mice with No. of liver metastases P*^a^* the ratio of liver-body

liver metastasis(n) Rang Median Mean (g/100g)^ƒ^

Control 8(9) 71(5-76) 39 35.7 4.64±0.28

HCS-L 6(9) 40(3-43) 22 19.4 0.043*^b^* 3.72±0.16*^b^*

HCS-H 5(9) 28(3-31) 17 14.5 0.029*^b^* 3.53±0.17*^b^*

*^b^*:P <0.05 by Mann-Whitney U test between HCS-group and Control; *^a^*:<0.05, by Kruskal-Wallis test among HCS groups and Control; ^ƒ^: Mean±SEM. P value<0.05 is significant statistically.
